# The Application of Neuromarketing Techniques in the Spanish Advertising Industry: Weaknesses and Opportunities for Development

**DOI:** 10.3389/fpsyg.2020.02175

**Published:** 2020-09-03

**Authors:** Miguel Baños-González, Antonio Baraybar-Fernández, Mario Rajas-Fernández

**Affiliations:** Facultad de Ciencias de la Comunicación, Universidad Rey Juan Carlos, Madrid, Spain

**Keywords:** neuromarketing, market research, advertising, consumer behavior, cognitive responses, marketing, neuroscience, neuroeconomics

## Abstract

Neuromarketing has aroused great interest in scientific research about consumer behavior and, consequently, in the advertising industry, which is searching for an alternative to traditional techniques for measuring efficacy. However, despite its development in the academic world, in the professional sector, its use is still very limited. The aim of this work was to find out the perception of advertising professionals as regards neuromarketing techniques for identifying its advantages and disadvantages compared to other research techniques, as well as the reasons why the use of neuromarketing is so much lower than expected in the Spanish market. The technique chosen for data gathering was the semi-structured interview, which made it possible to go into more depth with the subjects that came up. The interview, after a pre-selection of 100 professionals who matched the set criteria, was carried out individually, face-to-face, with a sample of 30 people with considerable professional experience in the field of marketing, sales communication, and market research; all of them belonged to leading companies from the sector. Once the information had been categorized into units with common themes, the results obtained were interpreted to explain how neuromarketing techniques are being used in the field of advertising. The results obtained led us to the conclusion that, even though neuromarketing provides more objective data and it is closer to what really happens to consumers when they are exposed to an advertising message, the ignorance of its true possibilities, the inability of suppliers of these techniques to transmit the value they add to research, its complexity, its high cost and time requirements explain why it has had scarce use in advertising research. The results revealed the real reasons for the rejection of neuromarketing techniques which, in turn, will make it possible to introduce both technological and formative solutions, allowing it to be incorporated into future research designs.

## Introduction

One of the main objectives of research into marketing communication is to measure its efficiency. For a long time, there has been a wide variety of measuring techniques and instruments used to better understand consumer behavior and the effectiveness of advertising messages although, as Winer (2011) points out, all have certain limitations and share considerable bias which makes full and accurate knowledge of consumer thinking impossible to obtain. Among other aspects, it is worth highlighting the large dependence on the will of consumers and their ability to describe their levels of attention, emotions, preferences, and future purchasing behavior in relation to an advertising campaign which they have been exposed to previously (Pozharliev et al., 2017). Another aspect to be considered is linked to the deficiencies when it comes to analyzing the motivations and processes related to the how and why of consumer decision-making (Winer, 2011). In addition, by basing results on indirect inference, to the subject bias of the observer is added the bias of the experiment itself, for example, peer pressure or other psychological or sociological phenomena pertaining to the individuals who make up the sample group all of which has a negative influence on the efficacy of the research (Wei et al., 2018).

Neuromarketing, as a discipline which uses the techniques of neuroscience applied to marketing, is closely linked to the new paradigm related to what is called the “experience economy” (Pine and Gilmore, 1998). In it, the emotions play a key role and also the theory of the mind, as set out by the neuroscientist, Damasio, who suggests a coming together of neurobiology and thought: “the formation of the mind is based on interactions between the nervous system and its organism” (Damasio, 2018a, p. 48). Research into the effects of advertising messages, level of acceptance or rejection, and the consumer’s decision-making can, on occasions, be difficult to express in words. Within qualitative advertising research, the advances in neurosciences in the last decades have opened new channels in the area of market research since they can “provide insight into the consumer’s mindset that traditional marketing test methods cannot offer” (Singh and Jain, 2018, p. 102).

As expressed in Simon’s theory of bounded rationality, our cognitive limitations, when it comes to information and time, make us to take decisions which are partially irrational, seeking “levels of conformity rather than maximum utility” (Simon, 1995). This model has been developed by other scientists who have gone into more depth in the knowledge of these cognitive biases which characterize humans. Among others, it is worth highlighting Kahneman for his revolutionary perspective on how our brain thinks, where “intuition and reasoning are alternative ways of resolving problems” (Kanheman and Tverski, 2000, p. 23) and on decision-making by two systems, one fast, intuitive, and emotional and the other, slow, deliberate, and rational (Kanheman, 2012).

Approximately 95% of mental processes take place in the non-conscious mind, which is precisely where the mechanisms which condition decisions are found (Braidot, 2005); in addition around 90% of our purchases are impulse buys and 95% of purchasing decisions are organized in the subconscious area of the brain (Álvarez del Blanco, 2010). Marketing specialists need to understand these cognitive processes which take place in the consumer’s mind to satisfy needs and design their communication strategies accordingly, however, the fact that they are unconscious, means it is difficult to express them (Shahriari et al., 2019). Faced with this situation, neuroscience suggests a wide variety of methods and tools to better understand the perception and behavior of the general public (Brenninkmeijer et al., 2019). The biological reactions and those of the brain, to marketing stimuli, are registered and can then be quantified and used for prospecting. The possibility of providing accurate and real data allows a better understanding of human psychology and how emotions influence decisions (Gani et al., 2018). Scientific advances in neurophysiology allow us to establish a taxonomy dealing with bodily changes and the emotions which cause them. That confirms the Jamesian theory set out by James, one of the fathers of modern psychology at the end of the 19th century, who linked each emotion directly to the corresponding bodily sensations that it caused (Pineda, 2019); on the other hand, at the end of last century, Damasio used empirical evidence to show how that, without emotions, or with certain dysfunctions in the emotional system, it is impossible to reach sensible decisions (Damasio, 2018b).

For those who defend neuromarketing, these research studies have the potential to go far beyond traditional focus groups and to be much more profitable by extracting data from the consumer’s non-conscious/subconscious (Nemorin, 2017). This is because neuromarketing seeks to find patterns of activation of brain circuits while receiving persuasive messages, in order to predict human behavior in future situations (Suomala, 2018) no matter which brand content is chosen (Nuñez et al., 2020). In other words, neuroworking studies the responses of the brain to marketing communications and the adjustment of those messages based on the feedback received with the aim of obtaining better replies (Ghorpade, 2017), without the conscious participation of the subjects, thus providing objective and scientifically measurable, emotional, and cognitive results for consumers (Cuesta et al., 2018).

However, despite all the advantages which, apparently, come from the techniques of neuromarketing as opposed to traditional ones from advertising research, its use is far from common and has even been the object of considerable criticism. As far back as 2004, the journal *Nature Neuroscience* dedicated its editorial in its July issue to this subject, warning of the power of neuromarketing to get into people’s minds, and alluding to the ethical questions thrown up by this type of practice. Among other comments, it questioned the morality of researchers who carried it out and expressed doubts about the scientific validity of the actual content behind the seductive appearance of ultra-processed colorful images of the brain’s activity.

In addition, neuromarketing also comes in for criticism because of the practical challenges of its application (Ariely and Berns, 2010). Among others, are: the ethical and legal issues (Pop et al., 2014), the difficulty of access to the necessary technology in certain countries (Brammer, 2004), running times which, on occasions are incompatible with the tight schedules which often prevail in the areas of marketing and advertising (Spence, 2019), or the need for expensive and complex equipment (Lee et al., 2007). Wei et al. (2018) set out some limitations of these techniques linked to bias caused by the experimental nature, which provokes a very different mental state in the subject from the normal one; they also refer to the size of samples and their reduced representativeness, warning that it is only possible to infer the impact during or soon after the launch of the campaign and that is somewhat risky with so much of the budget already invested. Another fact to bear in mind is the complexity of the reports based on scientific aspects which are far removed from the experience of most users and which require, moreover, elaborate interpretations for reaching conclusions and providing useful information (Stipp and Woodard, 2011). Varan et al. (2015) point out that the suppliers in this industry, in a constant state of evolution, offer a confusing range of different methodologies which are often patented. These authors show that those that sell those technologies have cultivated the expectation that their measures are more reliable than traditional methods because they measure the neurological and biological processes. The results of studies question those claims and suggest that advertisers should choose their provider carefully, with a certain skepticism about the trustworthiness and validity of the methods. If the results were truly error-free, the data obtained with the equipment from different providers would not differ from one to the other since they would be measuring the same variables. However, it has been shown that there is considerable disparity between the results recorded by different suppliers. In line with this last question, Brenninkmeijer et al. (2019) have reached the conclusion that providers of neuromarketing do not inform about the real experiments which they carry out or they inform in part, because the only material that we can check is that which they choose to share – the rest is a corporate secret which, effectively, means that there is no information to verify the validity of the different tools.

According to Global Ad Trends (2019), forecasts placed advertising investment for 2020 at around 660,000 million dollars, 7.1% more than in 2019. That estimation will probably be affected significantly by COVID-19. That considerable amount of money should be accompanied by another proportional investment to ascertain if the communication is really efficient. The commercial research sector in Spain had a net business figure of 513 million euros and, despite being in a constant state of expansive evolution over the last few years, the forecasts before the pandemic were estimating close to zero growth (Insight Analytics i+a España, 2019). From the point of view of business, we can consider investment in neuromarketing residual, since data show that in the professional field, it is far from being the most used – of the nearly 46,000 million dollars spent on market research, on a worldwide scale 81% is spent on quantitative research. In Spain, the figure is 84.6% (El publicista, 2018) and only a small percentage – the remaining 15.4% – is spent in the other area.

On the other hand, research into neuromarketing in the university field has followed a very different path. Starting a few years ago, it has become an emerging discipline as “one fourth of all Spanish universities are carrying out research about neuromarketing in Spain” (Andreu et al., 2014, p. 156). In the academic sphere, studies using these techniques have been profuse and able to demonstrate relevant qualities such as the relationship between its use and more effective communication which, in turn, has been influential in increasing sales (Ghorpade, 2017); also, its use in the creation of campaigns has led to greater accuracy and reaching the communication objectives of brands (Martínez-Fiestas et al., 2015). An additional aspect, are its possibilities in fulfilling one of the most desired purposes of any company or organization anywhere in the world to understand, predict, and change the behavior of their customers, all with the goal of designing more effective action (Spence, 2019); also, recognizing its potential for improving the practices employed by traditional marketing (Lin et al., 2018). The numerous academic publications cannot be considered indicative of the social mark left by these techniques as the impact of those texts is limited to the influence of the research in the academic field (Rodgers, 2018), and they are contradictory to the volume of business they generate in the industry of market research. Also, in the academic sphere, formulae are being sought to connect the academic world to professionals – the fundamental to the practical – using the presentation of research studies, which can be applied to the marketing industry (Lin et al., 2018); Suomala (2018) outlines a series of interesting proposals for cooperation between both areas in order to create optimum marketing campaigns through the use of neuromarketing.

Faced with the advantages and restrictions which any social research technique brings up – both the more traditional ones which have been in use for decades and the newer ones – Shen and Morris (2016) suggest that to improve the efficiency of advertising research, as long as they are sufficiently proven, both should be integrated into the same design. Marketing researchers should see neuromarketing, not as a way of replacing the traditional methods and models but as something complementary which helps improve our grasp and interpretation of human behavior (Cherubino et al., 2019). In this way, the shortcomings of the techniques which, traditionally, have been used the most and which are assertive by nature, may be compensated by neuromarketing techniques which are more objective and precise – the combination of the two can improve advertising efficiency (Ford, 2019). Baraybar-Fernández et al. (2017) published a piece of work in which they used neuromarketing techniques to record the emotional response of the subject, and questionnaires to measure the recollection of the message. This complementarity is a breakthrough in our understanding of the complex world of the emotions, one of the most persuasive arguments used in advertising messages today, as it dodges the problem of measuring its effectiveness in the receivers of the message, among other elements, due to the difficulty they sometimes have in verbalizing their feelings. Meyerding and Mehlhose (2018) state that, although it is unlikely that, in the near future, the methods of neuroimaging will be less expensive than other, traditional techniques, they identify its potential to establish a more efficient relationship between utility and cost, given that neuronal activity and other physiological signs in buyers contain hidden data regarding true preferences; thus, the high acquisition cost of neuromarketing studies would be offset by the usefulness of the results obtained.

## Materials and Methods

This piece of research has, as its goal, to discover the state of neuroscience techniques in Spain as they are applied to market research. Even though there are already quantitative data regarding the use of different research tools, no study has been carried out to obtain in-depth knowledge about the perception and experience of marketing communication and marketing research professionals regarding these techniques (Sutil, 2013). Our research which is, by nature, exploratory, uses a qualitative approach to gain insight into the situation, by means of the experience and opinions of a group of experts in communication, marketing, and research.

According to Taylor (Taylor et al., 2016; “Qualitative research is concerned with the meaning people attach to things in their lives,” p. 7), dealing with reality through the particular experiences of subjects in order to understand how they see that reality.

Consequently, the aim of qualitative research is to “reconstruct” reality as observed by the players in a previously defined social system (Hernández Sampieri et al., 2010, p. 9) without any manipulation by researchers. The definition of any specific reality comes from the different visions, opinions, and actions of interviewees.

### Objectives

The overall objective of this research is to find out the perception which experts, in different areas of marketing and communication of the specific Spanish advertising field, have of neuromarketing techniques.

The data tell us that the use of neuromarketing techniques as applied to market research is quite limited; however, looking beyond percentages, our aim is to understand why this happens and what motives there are for choosing more traditional methodologies in this type of research. The results and conclusions can be extrapolated, with certain caution, to other contexts similar to the Spanish.

The specific aims of the objectives of the study are:

- To understand the reasons why the real use of neurmarketing techniques is far below that of other techniques in the context.
- To identify the main advantages and disadvantages which the sector associates with neuromarketing techniques.
- To explore the possibilities which marketing and communication foresee for these research techniques.
- To find out the perception which professionals from the sector have of the processes of innovation and development of neuromarketing from an international perspective.

### Method

The technique chosen for data gathering was interviews; one of the most popular methodological tools for qualitative research in Social Sciences (Ulloa Martínez and Mardones Barrera, 2017), which is characterized by its flexibility and open style and which tends to focus on the personal experiences of interviewees rather than general opinions and beliefs (King and Horrocks, 2010).

For Taylor et al. (2016), the interview is a means of social interaction; understanding that can help the interviewer give meaning to the information they gather, as the way in which people answer also depends, to a certain extent, on how they think the interviewer sees them. According to those authors, what makes qualitative in-depth interviews different is that they make it possible to understand how people construct their realities – how they see, define, and experience the world.

Semi-structured interviews have been used – these are based on an outline of subjects or questions which the interviewer can modify by bringing in additional questions to pinpoint or extend data or information. In this way, not all questions are predetermined (Hernández Sampieri et al., 2010).

The semi-structured interview was chosen because, building on the initial questions which gathered the key subjects or areas of research, it offers freedom to go into more depth in topics which may come up, modify some questions based on the replies of interviewees, etc. This has meant it is possible to gain a better understanding of the state of neuromarketing techniques in market research.

In this case, in order to reach the objectives stated above, the following criteria have been established to obtain the opinions of interviewees:

- Knowledge and experience in the use of this type of techniques.
- Motives as to why the interviewee believes neuromarketing techniques are, or are not, used in market research.
- Neuromarketing techniques’ capacity for trully measuring the efficacy of sales messages.
- What are the advantages and disadvantages of neuromarketing techniques?
- Objectivity of the information obtained using these techniques.
- The future of neuromarketing applied to market research.
- The situation of Spain compared to other countries of our zone in the use of these techniques and its innovation capacity in this field.

The interviews were carried out individually during the months of March and April of 2020: although the original idea was for them to take place face-to-face, only the first could be carried out due to the measures introduced as a consequence of the COVID-19 pandemic, which meant we had to resort to IT tools, in our case, the use of Microsoft Teams. As indicated by Trindade (2016), nowadays, with technological breakthroughs in the field of communication, the way people are brought together for interviews is undergoing changes. Tools, such as videoconferences or the 4G system, are being brought into the design of qualitative methodology without losing the form of dialog which is characteristic of the interview; this means of obtaining information means it is still possible for the interviewer to see the interviewees (Orellana López and Sánchez Gómez, 2006) which means the interviewer and interviewee can maintain direct simultaneous contact during the interview.

The planned duration of each interview was 1 h and each one was recorded (permission having been previously requested and granted by the interviewee) in Microsoft Teams.

### Participants

The aim of this research was not to generalize the results obtained but rather to better understand, by means of opinions and experience of interviewees, the use of neuromarketing in advertising and marketing research in Spain.

Morse (1995) states that in qualitative research, there are no guidelines for estimating the most appropriate sample size as is the case in quantitative research. For this author, in qualitative research, you have to accumulate data to the point of saturation, which is the key to excellence in a study of this type; saturation is reached at the point where data gathering no longer supplies new information. Saunders et al. (2018) identify four saturation models in qualitative research: one is data saturation which is related to the identification of redundancy in the content obtained in the study. Sandelowski (1995) puts forward the concept of “information power” according to which, the more information contained in the sample group (as long as it is relevant to the study in hand), the fewer the number of subjects needed.

For the selection of the sample group, we bore two aspects in mind: having enough participants to allow us to reach the planned objectives and that those individuals should be directly involved in the process of market research. For the latter, we chose participants involved in: decision-making for choosing the research tools used, the application of those tools, and individuals whose work is influenced by the results obtained in those research projects. On the other hand, we looked for as much variety as possible within the sample group (Patton, 1988) with the goal of including different points of view and ensuring diversity of perspectives in the area of market research.

Based on these criteria, during the interview preparation stage, to choose the participants of the sample, we identified around 100 people with professional experience in the management of advertising research and sales communication in leading companies, which were related to the object of the study, from different areas linked to market research. This list was made up of advertising company directors, experts in market, and audience studies, as well as different professional profiles from advertising and public relations agencies. For the definitive choice of members of the sample group, 35 participants were contacted, based on their relevance to decision-making, market share in the sector, and the multinational nature of the company. Five of them decided not to take part in the study; for that reason, it was decided that 30 interviews would be carried out and we checked if it would be enough for data saturation. If not, new participants would be brought into the research since, in this type of study, the size of the sample is only known when the gathering stage has finished (Martínez-Salgado, 2012).

In view of the results obtained, it was decided that only the 30 pre-selected participants, 21 men and 9 women, would be interviewed as a depth and wealth of knowledge had been obtained from then so as to guarantee the data saturation criterion for the research.

On the other hand, in the sample selection, we opted for people with extensive experience in the area of communication in companies and organizations, managers who make decisions. That gave us an insight, not only into the experience of the participants in their current position, but also their aggregate knowledge from their long professional career.

In the end, the sample group of respondents was made up of 30 people who were divided into four groups depending on their place in the advertising system:

- Advertising and public relations agencies. Interviewees who had experience in some of the main national and international groups.
- Advertisers. This group was made up of people from some of the major advertisers at both a national level and an international level.
- Research centers. Professionals with experience in bodies specializing in market research.
- Media. Representatives of the main Spanish communication groups have taken part.

The average number of years’ experience in the area of sales communication and market research is over 15.

### Analysis

Finally, in qualitative research, analysis and interpretation of the information takes place based on the data obtained from the interviewees. The essential process of analysis consists of – using the completed interview – building up a wide variety of data which are unstructured and which, in this process, must be structured (Hernández Sampieri et al., 2010).

To carry out an exhaustive analysis of the material gathered, the first step was to transcribe all interviews as a preliminary stage to the definition of categories.

Once all recordings and transcripts have been checked, the next step was to categorize the information obtained, dividing content into categories in order to classify the data in units of common topics. In this process, we have reduced the quantity of information, which implies selecting, focusing, and abstracting data into units by their meaning, called “content categories,” according to specific thematic criteria (Massot Lafon et al., 2009). When defining categories, data are grouped, common information in interviewees’ discourse is identified, etc.

In this study, data have been organized into different categories according to thematic criteria which match the objectives defined in the design of the research:

- Reasons why the use of neuromarketing in market research is not more widespread.
- Neuromarketing techniques’ capacity for truly measuring the efficacy of sales messages.
- Advantages of these techniques over traditional tools.
- Disadvantages of these techniques compared to traditional tools.
- The combination of techniques for improving research efficacy.
- The market perspective of neuromarketing.
- The local and international vision of neuromarketing.

Finally, we carried out an interpretation of the results obtained in order to give an explanation of how the techniques of neuromarketing are used in the field of communication and market research, based on the opinions and experiences of professionals from the sector.

## Results

In our methodology, we defined a series of categories where the interviewees’ answers were placed. In this section, we include a description of the most relevant topics as expressed by interviewees in each category.

### Reasons Why the Use of Neuromarketing in Market Research Is Not More Widespread

Most interviewees agreed that, in the field of market research, neuromarketing techniques are used little or very little, among other reasons, because their results have not been shown to be definitive – which is what companies are interested in. In addition, a lot of the research has been carried out in universities rather than the professional world and academic activity differs considerably from that of the business world.

Another reason which explains the scarce use of neuromarketing is the ignorance as to the opportunities it offers; as stated by one director of a multinational advertising company, “professionals are not aware of these techniques and only large companies know how to interpret them.” This means that, even before the sales pitch, “you have to spend most of the meeting teaching,” claims a neuromarketing specialist. For one interviewee, with decades of experience in large advertising firms, the situation is even worse in small businesses: “they do not even know it exists, it is not even on the table, whereas traditional techniques are.”

Together with this problem is the inability of suppliers of these techniques to communicate the value they add to research, with one neuroscience specialist stating that, there is a lack of seriousness and an excess of “misinformation, which is not the same as a lack of information (…). They create confusion in the market and they have no idea.” This point of view is shared by advertisers, as the results obtained are not very direct and must be explained by someone – which may take away from their credibility: “It is not easy to explain what the benefits are to the people who take decisions and decide the budget.”

One interesting aspect which appears in several interviews is that, for these techniques to be used, the advertiser must ask for it, particularly in the case of international clients; the agency does not agree with these techniques and rarely decides to use them because it prefers to focus on its experience and intuition; the result is that “creative directors from big companies do not want people interfering and small firms do not have enough time and money to try it out.”

Another recurring feature of several interviews is the lack of interest in the sector because it is so new – they prefer to go to traditional methods or techniques and the way it has always been performed. This is because, as one director of an advertising agency pointed out, “the market has its habit of qualitative and quantitative criteria, these techniques are tried and tested and professionals know how to interpret them and they are easier to sell to clients; neuromarketing requires training to find out how to interpret data and sell them”; for that reason, from the point of view of clients, “things tend to be done as they have always been done because change requires effort” and “clients are reticent to innovate… (…). So when neuromarketing is made known, fear of change means things continue to be done the same old way.” We also observed this argument in agencies: “most companies are either traditional or do not really understand the results so they do not take the risk of using neuromarketing,” although they do also admit that they are complex techniques and, even though they are set out as something very attractive, “they have been left in no-man’s-land because the people who favor research do not see it as being rigorous enough and, for those who do not have a research background, it is too sophisticated for what they do.”

Finally, the complex state of the communication sector is also cited “for these innovations…rhythm is tremendously fast, ‘immediate’ is a must, and companies do not have time for novelties,” says an executive from one of the largest Spanish media groups.

Contrary to the majority opinion, some of those interviewed feel that neuromarketing is being used quite a lot in market research: they are professionals who work in research centers and their main argument is the ability of neuromarketing to measure the emotions which is what is raising most interest among communication and marketing professionals: “in Spain, there is a tendency toward, and a great interest in, emotions, and neuromarketing is the best way to measure them.”

### Advantages of These Techniques Over Traditional Tools

The advantage which stands out most, and the one on which most participants of the four groups of respondents agree, is that they are techniques “which distance themselves from the declarative and bring us closer to the response which is really produced by the impact of advertising,” going – in terms of decision-making – from opinion to certainty. Since it is not a declarative technique, neuromarketing is closer to what really happens with consumers when they are exposed to a sales message; there are no questions so the subject cannot think of an answer. What neuromarketing measures is, as one agency interview graphically explains, “… pure impulse. The person feels while they are asking for something, without thinking, minimum reasoning. That reaction is unpredictable, and I cannot hide it.” In the case of traditional techniques, as one advertiser said, there are “risks related to the ‘I cannot express’, ‘I do not want to admit’, ‘I cannot really rationalize what is happening’, it is a whim (…). And that is what neuromarketing helps to resolve.”

One of the advantages which stands out is its ability to predict behavior. This does not occur in the case of traditional techniques because “the fact that you can remember something does not necessarily mean that you are going to buy it,” whereas neuromarketing “goes one step ahead and provides predictability.”

It is also noteworthy that only a few sales messages overcome barriers to attention; if you do not get a person’s attention, you will not get anything and, as one advertiser points out, “neuromarketing understands which messages overcome the hurdles and can activate certain behavior.”

One interesting advantage is neuromarketing’s ability to justify the choice of more daring messages, as “creativity scores badly in any old-school pre-test; without neuromarketing techniques, that which is surprising, novel and shocking is penalized, whatever is outside the conventional – and that has been shown to go against efficacy because ‘conventional’ is useless in advertising.” Nowadays, most of the research which is carried out to assess the efficacy of messages provides results that are ambiguous and not very realistic and “afterwards, when analyzed using artificial intelligence or neuromarketing, a very different reality can be seen.”

One aspect which stands out is the depth of knowledge about the consumer which neuromarketing provides, as it gives information about the non-conscious part of participants and that is the key to behavior. This is of particular importance at a time when the consumer is changing so much. Neuromarketing, for an advertiser, “makes it possible to know the internal motivation of the customer, for the next action which the brand is going to take (…). That leads to greater efficiency with the budget.”

For some respondents, one of the advantages is its attractiveness because emotions “are fashionable” and these techniques, “even though they generate some rejection, they are very appealing because you want to know how the brain works, and about human behavior, and emotions: and it has been discovered that emotions are very powerful when it comes to selling and advertisers want to measure them using neuromarketing or quantitative tools, artificial intelligence….”

Finally, another advantage of neuromarketing is its objectivity, essentially because it removes the “mediatization” of the researcher, “it focuses on the real reaction of the subject” to a stimulus, in the words of a neuromarketing expert, “because it lets us see the variables which are activated in the brain of the subject when exposed to a stimulus and we discover – with total accuracy – the relationship of this individual to the brand”. However, despite a positive evaluation of the objectivity of data, one of the main disadvantages is related to the interpretation of data, as we shall see in the following category.

### Disadvantages of These Techniques Compared to Traditional Tools

We have just seen how objectivity is an advantage of neuromarketing techniques; an objectivity which, as one director of an agency points out, is limited by the interpretation of the researcher, “it is more objective, but with many pitfalls because data are more objective, but then comes the interpretation.” Having said that, as one interviewee from a research center noted, interpretation is a general problem because neuromarketing “when it comes to interpretation, it is done personally, like with any other technique.”

Another problem of neuromarketing, which appears in several interviews, is the context of the tests; even though, as one advertising agency director says “it is not much worse than in any of the traditional techniques,” it is one of the most repeated disadvantages: “an unnatural and highly controlled environment. You take people out of their natural surroundings and they pay more attention to the technology because of the artificial environment”; according to a director of one multinational advertising company. It is such an unnatural context that it conditions the whole research process. It must be borne in mind that, as is highlighted by one market research expert, this is all normally performed in a laboratory with all that entails. That point of view is qualified by another research expert when they state that technical teams are getting smaller and smaller and some techniques can now be applied outside the laboratory in a simple way.

Another disadvantage is the complexity of some neuromarketing techniques in that “there is a great dependence on technology and software for which we do not have total expertise,” particularly that some techniques are highly invasive such as “resonance” which, in addition, requires a considerable degree of interpretation. Some of these techniques cause anxiety in people by surrounding them with machines in a situation which could be likened to medical tests – that causes a greater bias than that which occurs in traditional techniques, precisely due to the “laboratory effect.”

One advertiser suggests that the profile of researchers who work with neuromarketing is too scientific, “which makes it difficult to translate to non-scientific profiles” and places the genesis of the problem in the terminology used by researchers.

Added to all of that is the lack of a common standard in the market. For one expert in research “each company uses its own names and speaks about different things” and that leads to great confusion, and, on occasions, to work with “indicators which are different, even though they might have the same name, measuring different things.” Working like that it is impossible to reach the same conclusions using technology from different suppliers. At this point one sees “a certain retrogression to protect a sector which seeks to prolong the mystery.” As one neuromarketing says, when the discourse is not reliable and does not coincide with the “results, it generates mistrust; there is some sort of magic box which has not been controlled and reliability wanes” being seen as a lack of transparency.

There is also a repetition of several problems which have to do with costs, sample size, and the time needed to apply these techniques. Some interviewees from research firms play down these inconveniences arguing that: they are not always more expensive, the depth, and repercussion of the results compensate a possible extra cost, or, the samples from some traditional techniques do not produce generalizable results either; however, the perception of interviewees is that the procedure with neuromarketing, as summarized by one director from an advertising agency, is “slower, more expensive and it is difficult to obtain a broad sample group.”

One feature which produced a difference of opinion among interviewees was the possibility of comparing results with the past; so, for one of the respondents “not enough studies have been carried out and there is not a comparison with the past; the sector has no benchmark, nor has the brand nor the campaigns…” for another “objective measures already exist; there is a benchmark for comparing with other actions or campaigns.”

Another interesting aspect, again with contradictory opinions, is neuromarketing’s ability to work by and of itself in market research. We will deal with this point in greater detail later in one of the other categories of analysis.

### Neuromarketing Techniques’ Capacity for Truly Measuring the Efficacy of Sales Messages

This category was the one which threw up the biggest discrepancies among interviewees, fundamentally because of the diversity of perspectives from which advertising efficacy can be approached; in the words of one interviewee from a research center, “efficacy is a broad term and with neuromarketing alone it cannot be measured” and, along the same lines, another respondent from the group stated “the doubt lies in what we call ‘efficacy’ (…). Depending on what is being measured, in some cases efficacy is measured.” For that reason, answers go from “definitely not” to “100% yes.” However, most people acknowledge that certain aspects which are associated with advertising efficacy can be measured if conditions are appropriate.

Regarding the appropriate conditions, the expert in neuromarketing who claimed that these techniques can measure advertising efficacy 100%, did clarify that this happens “if you have teams and neurologists with marketing experience. That does not mean that those who are selling it now are able”; and for one advertiser “they could really measure the advertising efficacy, but it is not being used properly.”

The majority opinion is that neuromarketing can measure certain aspects of the message, but it needs to be combined with other techniques, a combination which has been considered in a category of analysis which we will see below. As one advertising director puts it, “efficacy, as the advertiser understands it, means purchases and along the road that leads to a purchase, there are a lot of important factors; with these results it is difficult to forecast sales”; along the same lines, an executive from an agency reckons that the emotional component influences the purchasing decision but, although “we are more compulsive than we think, we are not as much as neuromarketing thinks we are.” Measuring efficacy in marketing communication is a complex task and emotions are only a part of it, with more or less importance depending on the aims of the campaign. Using neuromarketing techniques we can ascertain if the message reaches the public at an emotional level, but its efficacy will only be measured if that is the objective of the campaign. If however, the aim is to retain the message, neuromarketing, as one interviewee from a research center stated, can be seen “to activate something related to the memory but not retention” – the subject has to be asked about that.

Some of the disadvantages explained in the previous category also surfaced here. As one director of a multinational technological company said, neuromarketing is considered “useful in some cases (but) with the limitations of the sample, the context … nowadays it is insufficient.”

### The Combination of Techniques for Improving Research Efficacy

In few sections of the research, we came across an opinion so commonly shared by the panel of expert interviewees. In fact, a significant number said they could only perceive the use of neuromarketing in conjunction with traditional techniques, both quantitative and qualitative. With the chance of designing hybrid models, they did not have to think twice about using terms such as “compulsory,” “recommendable,” or “totally necessary” when referring to integration. From the point of directors “it enriches the essential information for decision-making” and enables a global vision which avoids simplistic approaches in the understanding of how the consumer establishes their relationship with the company.” They understand that each procedure has its limitations and each one can provide valuable information to allow a more accurate vision of reality. The marketing director of one of the main television groups in Spain stresses this point: “in the end, traditional meters: frequency, coverage, or GRPs will continue; so, the instruments associated with neuromarketing must be added to existing research formulae those which are generally accepted and agreed to by the sector.”

Consequently, they do not consider the use of neuromarketing for individual projects except for very specific ones, and they acknowledge its validity as a complement in strategic decision-making. They are totally convinced that neuromarketing is not the answer to everything – something which was promised at the beginning. We have observed how neuromarketing has gone from being an attractive product which raised curiosity to being considered a reputable complementary research technique.

Most people favor its use in conjunction with other qualitative methods, such as focus groups, depending on the aims of the research. Among its strengths are, as we observed in the category of analysis, its ability to reduce the possible subjective biases associated with the classic formulae, based on declarative processes in which individuals state their opinions verbally. One director of marketing who was interviewed feels that “neuromarketing is a significant aid in analyzing behavior and identifying consumers’ behavioral patterns.” Consequently, the ideal thing is to integrate the “biological reaction of the spontaneous and the reflexive component, which is characteristic of prompted analysis.” “This direct interpretation of reactions to stimuli is insufficient for evaluating the effectiveness of any particular communication – other complementary methodologies which allow access to the conscious perception of the individual must be applied to be able to explain the effectiveness of any given element of advertising.” This is the opinion of a research manager of a multinational company in the technology sector.

There is a favorable opinion of the contribution of neuromarketing to these mixed techniques, as a mechanism for bringing us closer to the emotions and unconscious decisions. No member of the panel questions the relevance of the emotions in modern advertising messages and how hard they are to measure. As one manager of Kantar Media told us: “customers feel more comfortable with quantitative data and neuromarketing provides quantifiable indicators, making it a perfect complement for qualitative techniques.” However, despite there not being any doubt regarding coordinated applications, there is a considerable dependence on economic resources and time availability. This leads to it being commonly used by advertisers who invest heavily for worldwide brand campaigns, whereas its inclusion in the research activity of small businesses and as a one-off is more questionable. That means it will continue to be a support for creative intuition and have scarce qualitative research.

Its joint use is more highly valued when in association with developments related to machine learning and data science since, as one advertiser comments, “it is essential to mix quantitative and qualitative research and artificial intelligence in order to classify by personality”; and this harmonization “allows us to broaden our knowledge in aspects as interesting as brand loyalty.” With reference to that field, the director of one internationally renowned audience and market research company stated that: “digital advertising is basing itself on quantitative indicators, where the speed of obtaining data – almost the immediacy – is king, but these quantitative indicators must be combined with qualitative ones. That’s where neuromarketing (…) can help end the dictatorship of the ‘click’ and web analysis; now the ‘click’ and immediate response have the power (…); helping to go in-depth into the reasons behind human behavior.” One of the managers of a media agency provides us with the following interesting thought: “it is a case of a generational change, younger professionals are only interested in the quantitative” and, therefore, the future success of neuromarketing will be related to its ability to become part of big data, by incorporating biometric data co-aligned with geolocation and sales.”

### The Market Perspective of Neuromarketing

The general tendency favors a positive evolution in the development of neuromarketing. By contrast, there are divergences in terms of the speed and relevance of the process. The most significant group believes in a slow growth which could only be changed by the incorporation of a big player because in Spain, this field has been shared out evenly; for one research specialist, “if that situation did come about, neuromarketing would cease to be a tool used by leading pioneer brands who feel curiosity for any new technology which aids the process of continuous improvement, and it would extend quickly.” That forecasted growth could be under threat with the economic situation brought on by COVID-19, where a cut in investment spending would affect the whole advertising industry and, consequently, research budgets. Although, as one business manager from a multinational publicist says, “it could also become an opportunity for neuromarketing, with the need to assess the changes in consumers’ moods and how that influences their buying behavior, basically, discovering new insights.” However, there is a certain agreement when considering that, in the medium term, “it will end up breaking onto the scene and being part of research routines, although in the short term that will be more difficult because of price tension,” says an executive from one of the main advertising agencies.

The most pessimistic, despite not denying the potential, emphasize starting from scratch, building a new story, “even a new name,” mentions the managing director in Spain of a multinational from public relations. “This new story must be worded around a new focus coming from the hypotheses of attention economics and new consumption styles,” states the marketing director of a leading media player, “in our day-to-day, we know that the consumer does their buying, taking their decisions, while talking on the mobile phone.” Some of the interviewees from agencies specializing in neuromarketing services are also aware of this need: “we no longer talk about neuromarketing, we talk about conductual behavior economics,” a concept which deals with the study of cognitive biases in consumers’ decisions.

The future development of neuromarketing will depend directly on its ability to overcome the disadvantages which are perceived today and which were recorded earlier in this piece of work. “Until it can overcome cost barriers and cost-benefit shortfalls, it will remain secondary and a mere complement (…), at the moment, the return on costs is not what we had hoped”; according to one marketing director. On the other hand, there are also different comments which refer to the times of use and each technique in particular; “it will still be used more in the post-test phase than the pre-test phase,” is the opinion of several members from advertising agencies. Also, “not all techniques will progress at the same pace – the eye-tracker, the electroencephalogram (EEG), or magnetic resonance imaging (MRI) have considerable possibilities for future expansion,” says one specialist with extensive experience in neuromarketing techniques.

Another of the challenges relates to the simplification of data and KPIs, with a common theoretical model or framework more akin to the client’s aims. The standardization of meters would aid understanding and accessibility; “it has to cease to be like a magic box,” commented one of the respondents. This is particularly true if it can show its worth for predicting the market – that would accelerate its use. Along those lines, we share three comments as examples of the most common feeling among marketing directors “it should cease to be offered with an added value (…) in order to obtain personality of its own right,” “it will become more commonly used as long as it can demonstrate its relevance for obtaining customer knowledge and for improving the effectiveness of advertising messages; there is not enough evidence, results must be shown”; “there has to be a unification of criteria, not like now where each company defends its own tool, its business, and that causes great confusion.”

According to the group of experts in these research techniques, the process of improvement is also related to “the ability to go on learning from the advances in other scientific fields such as biology, health, knowledge of the nervous system, and informatics.” Regarding the latter, if developments in hardware and software continue being passed on to neuromarketing and it can escalate the data to come into synch with quantitative demand, there will be an acceleration of its promotion: “what is needed is an extension of samples in order to obtain statistical validity and its adaptation to real-life scenarios, it is the only way of increasing its specific value in marketing sciences.” Another interesting thought, put forward by a business manager from a multinational market research company, defends the future potential related to technological developments: “continuous data are a reality, thanks to follow-up using a mobile phone and they will reveal our mood, and the emotional disposition of the consumer.”

Even though “any client wants their ideas to be tested” and shows “concern to know how well markets identify with their brand characteristics,” according to the comments of interviewees – even those with a creative profile, it is also necessary to bear in mind that not all business categories have the same expectations. There are more traditional sectors and others, for example, those which are linked to leisure and entertainment will be more inclined to introduce these tools in their research activity. In the retail field “its potential for identifying the hot zones at the point of sale and measure consumer behavior when reacting to stimuli is incredible, making it possible to optimize those aspects related to communication and the consumer” – that is the claim of one international corporation manager with establishments all over the world. Likewise, the large platforms of e-commerce or service agencies of digital advertising and social networks, feel that its implementation is irreversible for the study of behavior and improving user experience.

To sum up, what we understand as “neuromarketing” will be part of a more holistic concept of consumer research, based on the acquisition of knowledge to better understand their behavior. An articulated vision with a double axis made up of the contextualized information of data, in order to bring them closer to reality and the needs as expressed by organizations, and its ability to construct standardized predictive models which adapt better to change.

### The Local and International Vision of Neuromarketing

Results reveal a widespread in responses, among other reasons, because of the scarce knowledge demonstrated by various interviewees of the situation of neuromarketing in Spain and the rest of the world. Another reason for the difference of opinions comes in the form of one of the respondent’s answers: “with there being so many small, good quality companies in so many different places, perception cannot be very specific”. There is no sensation of technological mastery and clear differentiation.

Many of the responses are influenced by their professional experience which, in turn, depends on specific projects and specific clients or, in the case of multinationals, those offices from the group in other countries which have often applied neuromarketing techniques. The business manager of a Japanese multinational from the advertising sector with its European head office in London, states that “in Japan, it is used regularly in campaigns; also in the UK and it will reach the rest of Europe soon, but it will take time for it to become part of the day-to-day.” One of the members of a renowned Spanish agency which is now part of a major holding group worldwide, acknowledges that “I have been surprised by the Italians and their reports and how they justify decisions using this type of technique (…), it is something which has not happened to me when working with clients from other countries.” Innovation in the area of neurosciences is also often associated with countries which are more advanced technologically and those which have taken the initiative in the development of marketing and management; one marketing director places the pioneers “in the Anglo-Saxon world, because it comes from there but, the thing is, the whole profession comes from there.”

It is, perhaps, because of this last reason, that the most named countries are the United States and the United Kingdom but the differences between them and countries like Denmark, Italy, France, Germany, Sweden, Netherlands and, depending on the interviewee, Spain, are minimal. It is interesting to note the presence of other countries which would not be considered references in advertising – Poland, Norway, or Belgium. The mention of other South American countries such as Brazil or Argentina, for the interest they show in these techniques, is also significant.

The perception of the situation in Spain is similar to the other countries of our environment when it comes to the supply of services, results, and innovation. Overall, there is satisfaction with the services provided by consultants. This is true of both clients: “we have a lot of contact with our colleagues in other countries and Spain is one of the places where neuromarketing techniques are used most,” and service providers: “Spain is a mature market which has made a lot of progress in research… I think we are in the top ten worldwide in research and neuromarketing techniques too.”

We have observed a link between the responses and the type of company where the interviewee works. Those who work in multinationals for global advertisers tend to have quite a positive vision of the level of neuromarketing in Spain: “as far as I know, the level of usage is the same everywhere (…) when we are talking at the agency, (…) I do not notice differences with other countries.” It would appear to be more a question of advertisers than geographic zones: “rather than countries, it is a question of the brands that apply these techniques at an international level; there are leading brands who which are the ones which are the ones who position themselves most in the area of emotions.”

## Discussion

With this piece of research, we have reached our main objective: to discover the perception which marketing and communication professionals have of neuromarketing techniques. By means of the interviews carried out we have been able to understand the current situation of neuromarketing in the professional field, as well as the advantages and disadvantages of this type of technique and its future possibilities.

The first conclusion of our work highlights the limited use of neuromarketing techniques and the ignorance of professionals of the real possibilities that these techniques offer for market research. The perception which interviewees have of neuromarketing is that its development is much greater in the academic world than the professional one, with a particular emphasis on the difference between research in universities and business; in that sense, Lin et al. (2018) had already expressed the need to connect the academic and professional spheres and Suomala (2018) highlighted the large production of research using neuroscientific tools in the academic community as opposed to the long road ahead to reach the point of general application in marketing action.

In that sense, certain specific barriers to the general application of neuromarketing have been identified – some of them were related to the general inertia of organizations and the tendency to do things as they have always been done. There is an innate reticence to the introduction of new research methods when there is a need to clarify the added value and, in addition, they have to compete with the pressure caused by the challenges and immediate problems faced by the companies in the sector. Most of them are immersed in meeting objectives and tasks which take up most of their attention – that makes it difficult to implement new tools.

In a context of so much competition and such urgent decisions, it is worth pointing out how difficult it is to understand some of these techniques and the lack of interest in developing measures which favor their incorporation into existing processes. That all requires an intellectual effort and learning time which does not tend to be available to directors, especially if the providers of these technologies are not able to communicate the added value which neuromarketing would provide for market research. Along those lines, Brenninkmeijer et al. (2019) concluded that the providers of neuromarketing do not inform about the actual experiments or they do so only partially, so that there is no information to verify the validity of the tools.

Skepticism is a way of rationalizing a reality which collides head-on with certain preconceptions which directors refuse to question until they have evidence and which, on most occasions, have their origin in the success of competitors when applying them.

The result is that many marketing and communication professionals feel uncomfortable with certain research designs which reveal their shortcomings or lack of experience; a situation which forces them to depend on the criteria of a series of specialists whose skills they are not able to judge. We have been able to check how, in effect, some neuromarketing practices are so complex and move forward so quickly that not even the experts interviewed could agree on their functionality. The accelerated growth of technology and specialization means it is more and more difficult, even for researchers, to decipher its possibilities. On occasions, they freeze when faced with the need to establish an agreed framework which governs these activities and their benefits when applied to the reality demanded by the market. Following what was stated by Stipp and Woodard (2011), this problem has been present almost since the outset of neuromarketing. Even at that time, in the reports of the results of studies, based on a complex science which is beyond the experience of most users, it was more difficult to achieve clarity and communicate the information than in traditional market research reports.

At the same time, the adoption of these solutions tends to require some or all of the following: a reordering of the budget priorities, an increase in expenditure, the creation or elimination of relationships with tested collaborators, losses, the assumption that there will be new competition, or a willingness to surrender what in psychology is called the “comfort zone.”

Other problems are identified which are not always justified, since more experienced interviewees in neuromarketing provide arguments which could refute those answers; in any case, bearing in mind that our aim was not to discover the real possibilities of neuromarketing but rather the perception which the sector has of it, the doubts it generates are relevant for our research. One noteworthy criticism is the context in which this type of tests take place – far from the natural setting in which subjects receive advertising messages. Technology is very present in this type of tests which are sometimes too similar to medical studies and all that makes the results obtained using these techniques less credible and realistic. The problem is the use of samples making it impossible to generalize results and also the time it takes to carry out these tests – normally far removed from the needs of advertising which, more often than not, requires almost immediate results. Along those lines, Spence (2019) includes what they consider practical challenges for the application of neuromarketing to commercial research. These include: cost, time, difficulties for accessing the necessary technology, or the context in which subjects take the tests.

One problem, linked to the lack of standardization of the measurements and the diversity of technologies offered by neuromarketing providers, deserves a special mention. This problem was highlighted by Varan et al. (2015) when they claimed that providers offer a confusing range of different technologies and suggest the advertisers maintain a certain skepticism about the reliability and validity of the methods. For the interviewees, neuromarketing throws up a series of important challenges for those who must decide on its use; in addition to scientific aspects, the necessary knowledge in order to analyze the results, or a dependence on technology, also, the confusion caused by providers with a complex offer of indicators, names, and methodologies that make it more difficult to break away from routines which have become the norm in market research. The potential of neuromarketing for measuring advertising efficacy can be perceived, but its limitations and the problems it generates outweigh the possible advantages it offers.

Despite these limitations, the interviewees also put forward important advantages, the first of which is that neuromarketing results do not depend on respondents’ results to a series of questions as is the case with traditional techniques. This question was pointed out by Pozharliev et al. (2017). This information aids the understanding of what really happens to consumers when they come into contact with an advertising message, providing a deeper understanding, directly related to the non-conscious part of the subject, making it possible to predict behavior.

One particularly interesting conclusion for advertising creativity is the ability which, according to some interviewees, neuromarketing has to assess the most creative, novel, and surprising messages. Everybody finds it difficult to explain in words which creativity is more striking and the answer tends to be in line with what the brand has always done. This is an important bias of the traditional techniques as responses are often influenced by the positioning of the brand and other previous messages known to the subject. However, with neuromarketing, there are no questions but rather a simple observation of the subject’s immediate reaction and that is how we discover which creativity is the most useful for the advertiser.

Along the same lines, neuromarketing is considered to be able to identify which messages can overcome attention barriers, that being the first hurdle for reaching any communication objective. Although reality may still be far removed from that vision if, as Wu (2020) states that the neuroscience of attention is still too primitive to explain attention on a large scale, although this author also points out that scientists have understood that the human brain has the incredible ability to pay no attention and that, if exposed for long enough, we can become indifferent to any stimulus. That makes it all the more necessary to know the ability of messages to capture attention.

Given that it is information which subjects have not declared, the objectivity of the data obtained by neuromarketing is worth pointing out. This is in line with the proposal of Cuesta et al. (2018) for whom neuromarketing provides objective and scientifically measurable results from the emotional and cognitive answers of the consumer. That reveals the need for professionalism and experience in the researchers who analyze the results obtained by means of neuromarketing techniques to obtain a valuable and accurate interpretation of the data.

Triangulation, or the combination of different methods or different types of data to improve the quality of the research and achieve greater depth from the data obtained, is very present in research. Although they do not employ this term, practically all interviewees coincide in highlighting the difficulty for using neuromarketing as the sole technique in market research, putting forward the need to combine it with other traditional techniques, developing what some call “mixed designs” or “hybrid models” whose greatest usefulness would be to eliminate the subjectivity which they consider a characteristic of affirmative techniques. This conclusion is in line with the proposals of authors such as Baraybar-Fernández et al. (2017) who have defended this type of combined designs or Shen and Morris (2016) suggesting that, in order to improve efficacy in advertising research, neuromarketing techniques and conventional ones should be integrated into the same design.

Even though the future brings up many question marks and there are too many variables which can influence the development of neuromarketing, the forecast is a positive progression, although it is difficult to give an answer about the pace of that evolution. If, several months ago, factors such as the appearance of new players in the market, economic circumstances, or the evolution of investment in advertising made it difficult to make any sort of prediction regarding neuromarketing, the evolution of the crisis caused by COVID-19 and the more than probable ensuing economic crisis make the future even more uncertain. The first challenge is its ability to overcome the different problems which the sector senses and to relate to and interact with other scientific fields.

In the future, neuromarketing will be part of a more holistic concept of research about consumers which will improve understanding of behavior. A vision articulated on a double axis composed of the contextualized information from data and the ability to construct standardized predictive models. Within this multi-technique design, there are some interesting proposals, for example, the combined use with developments related to machine learning, artificial intelligence, big data, glocalization, etc. The difficulty when comparing the state of neuromarketing in Spain with the rest of the world is also apparent. This is essentially because the responses are influenced by the professional experience of each of the respondents, which frequently leads to innovation in the area of neurosciences being associated with more technologically advanced countries and with those which have taken the initiative in the development of marketing and management. A relation can also be observed between the responses and the type of company where the interviewee works. The use of neuromarketing would appear to be more a question of advertisers than geographic areas. Whatever the case may be, the perception of the situation in Spain is similar, in terms of services on offer, results, and innovation, to the rest of our area.

Any marketing plan which attempts to project a future scenario must consider, among its priorities, a greater cognitive knowledge of their publics, directly related to the understanding of the processes of perception, feelings, emotions, will, conduct, and consumer communication. In the face of this challenge, neuromarketing proposes new ways which do not satisfy everybody. With the results of this research, we can know the real causes of the rejection of neuromarketing techniques for market research, thus making it possible to implement the technological and formative solutions which favor its incorporation into future advertising research designs.

We know what providers offer, and we can detect the potential of neuromarketing in market research, but it would be good to go on investigating to check if this offer really has so many problems – some of which as serious as the lack of standardization of the measures or the diversity of technologies – and if the perception which the sector has of these techniques is justified with the supply from different providers. It would also be beneficial to compare neuromarketing techniques with traditional ones in aspects as relevant – when it comes to choosing research designs for market research – as costs, time scales, or the context where they are carried out.

## Data Availability Statement

The original contributions presented in the study are included in the article/supplementary material, further inquiries can be directed to the corresponding authors.

## Ethics Statement

Ethical review and approval was not required for the study on human participants in accordance with the local legislation and institutional requirements. Written informed consent for participation was not required for this study in accordance with the national legislation and the institutional requirements.

## Author Contributions

MB-G, AB-F, and MR-F contributed to conception and design of the research. MR-F designed the interview. MB-G and AB-F interviewed the sample components for data collection. All authors analyzed and interpreted the data. All authors contributed to manuscript revision, read, and approved the submitted version.

### Conflict of Interest

The authors declare that the research was conducted in the absence of any commercial or financial relationships that could be construed as a potential conflict of interest.
